# Case Report: Venetoclax combined with hypomethylating agents for the treatment of newly diagnosed with mixed-phenotype acute leukemia and a literature review

**DOI:** 10.3389/fonc.2025.1693061

**Published:** 2025-10-27

**Authors:** Ruihua Mi, Shuli Guo, Weidong Yang, Lin Wang, Yixuan Ma, Lin Chen, Dongbei Li, Xudong Wei

**Affiliations:** ^1^ Department of Hematology, The Affiliated Cancer Hospital of Zhengzhou University & Henan Cancer Hospital, Zhengzhou, China; ^2^ Department of Hematology, Luoyang Central Hospital Affiliated to Zhengzhou University, Luoyang, Henan, China; ^3^ Department of Hematology, Anyang District Hospital, Puyang, China; ^4^ Central Laboratory, The Affiliated Cancer Hospital of Zhengzhou University & Henan Cancer Hospital, Zhengzhou, China; ^5^ Institute of Cancer Research, Henan Academy of Innovations in Medical Science, Zhengzhou, China

**Keywords:** mixed-phenotype acute leukemia, venetoclax, hypomethylating agent, treatment, literature review

## Abstract

**Objective:**

To investigate the efficacy and safety of venetoclax (Ven) in combination with hypomethylating agents (HMAs) for the treatment of mixed-phenotype acute leukemia (MPAL).

**Methods:**

From July 2023 to April 2025, 4 newly diagnosed MPAL patients treated with Ven combined with HMAs at the Affiliated Cancer Hospital of Zhengzhou University, Luoyang Central Hospital and Anyang Regional Hospital were retrospectively analyzed to determine the efficacy and safety of this treatment. The relevant published studies were reviewed.

**Results:**

This study included four patients (2 males, 2 females) with a median age of 47 years (range: 40–80 years). Three patients were classified as having B/myeloid MPAL, and one was classified as having T/myeloid MPAL. All patients achieved complete remission (CR) after one cycle of venetoclax combined with HMAs. Notably, Patient 3, who tested positive for the BCR::ABL1 fusion gene, received additional tyrosine kinase inhibitor (TKI) therapy. The median duration of myelosuppression during induction therapy was 26 days (range: 7–36). Patients 1 and 4 developed infections during induction, which were controlled with aggressive antimicrobial treatment and supportive care. In contrast, Patients 2 and 3 tolerated the regimen well without significant adverse events.

**Conclusion:**

The treatment of MPAL with Ven combined with HMAs achieved a high remission rate and can be used as an alternative treatment for MPAL.

## Introduction

Mixed-phenotype acute leukemia (MPAL) is a rare aggressive leukemia that accounts for 2~5% of all cases of acute leukemia among adults (1). The risk of death due to MPAL is 59% and 26% higher than the risks of acute lymphocytic leukemia (ALL) and acute myeloid leukemia (AML), respectively. For The current evidence suggested that MPAL patients ineligible for standard chemotherapy or allogeneic hematopoietic stem cell transplantation (allo-HSCT) have significantly poor outcomes (2). Therapeutic options for adult MPAL patients remain suboptimal, largely attributable to the high prevalence of adverse cytogenetic abnormalities (1). While Philadelphia chromosome-positive (Ph+) MPAL may benefit from the combination of tyrosine kinase inhibitors (TKI) with chemotherapy, effective strategies are notably lacking for the majority of non-Ph+ MPAL cases who lack targetable mutations. This significant unmet medical need underscores the urgency to explore novel therapeutic targets and more effective treatment regimens for this high-risk population.

Venetoclax (Ven) is a B-cell leukemia/lymphoma-2 (BCl-2) inhibitor that is currently widely used in clinical practice for the following AML patient populations: patients ineligible for intensive chemotherapy, patients with refractory/relapsed disease, and even newly diagnosed AML patients eligible for intensive chemotherapy. The application of Ven in patients with MPAL currently shows that it is more frequently used in B/myeloid MPAL patients. The treatment lines are generally initiated later, and various combination regimens are employed, often reported as individual case studies; for T/myeloid MPAL patients, the number of reported cases is even more limited, and the use of Ven in newly diagnosed T/myeloid MPAL patients has not been reported. In particular, Park et al. (3) reported the case of a T/myeloid MPAL patient who was given a vincristine, daunorubicin, L‐asparaginase, and prednisone (VDLP) regimen for 10 days and subsequently developed septic shock, for which chemotherapy discontinuation was needed. After the infection was controlled, bone marrow examination indicated that the disease had not achieved remission. The patient was then given 1 cycle of Ven combined with decitabine, and the patient achieved remission. Here, we report the cases of 4 newly diagnosed MPAL patients who were treated with Ven combined with a hypomethylating agent (HMA) and successfully achieved complete remission (CR).

## Case 1

**Figure 1:**
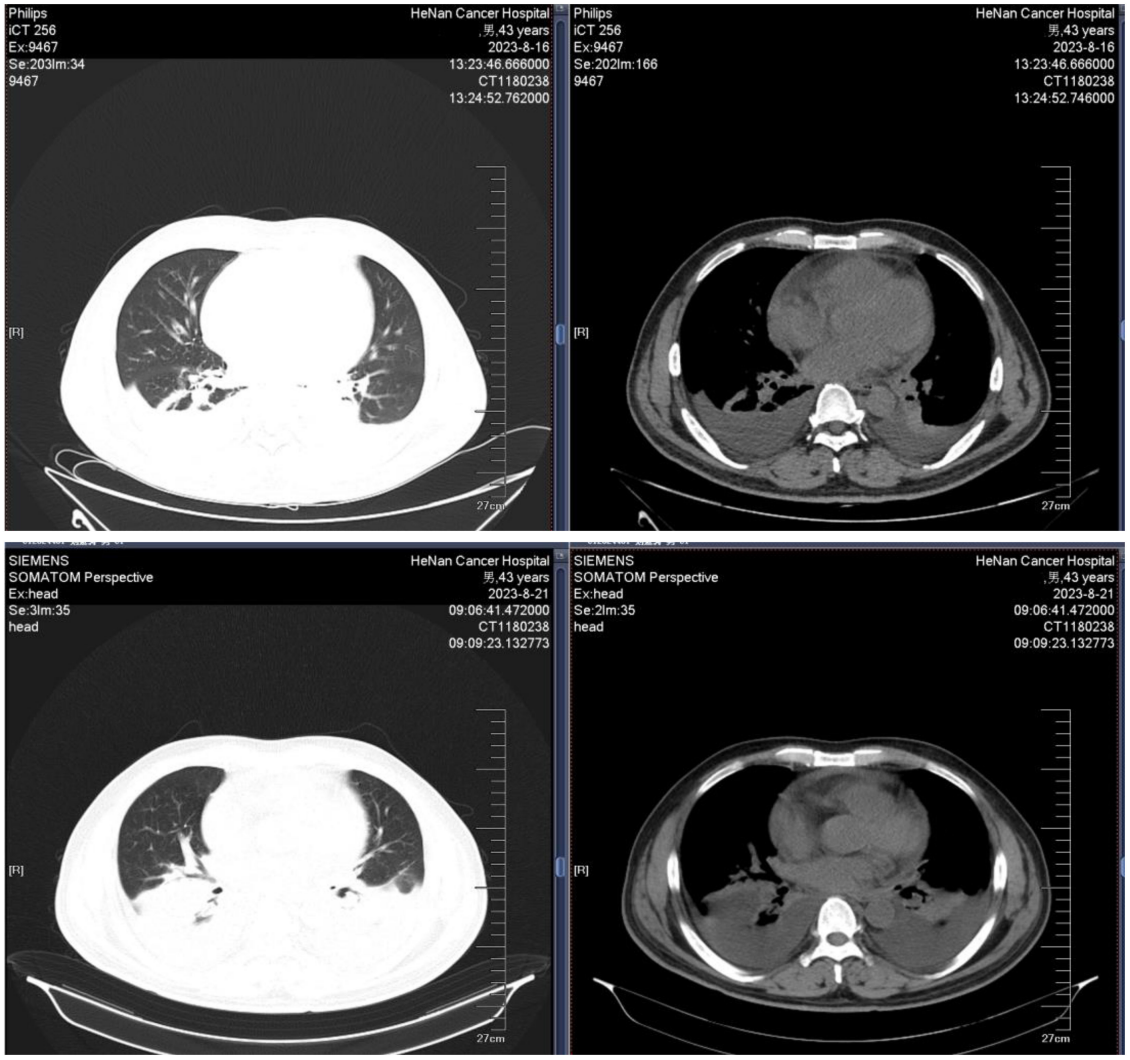
Example images of the chest CT during induction therapy in Patient 1.

**Figure 2:**
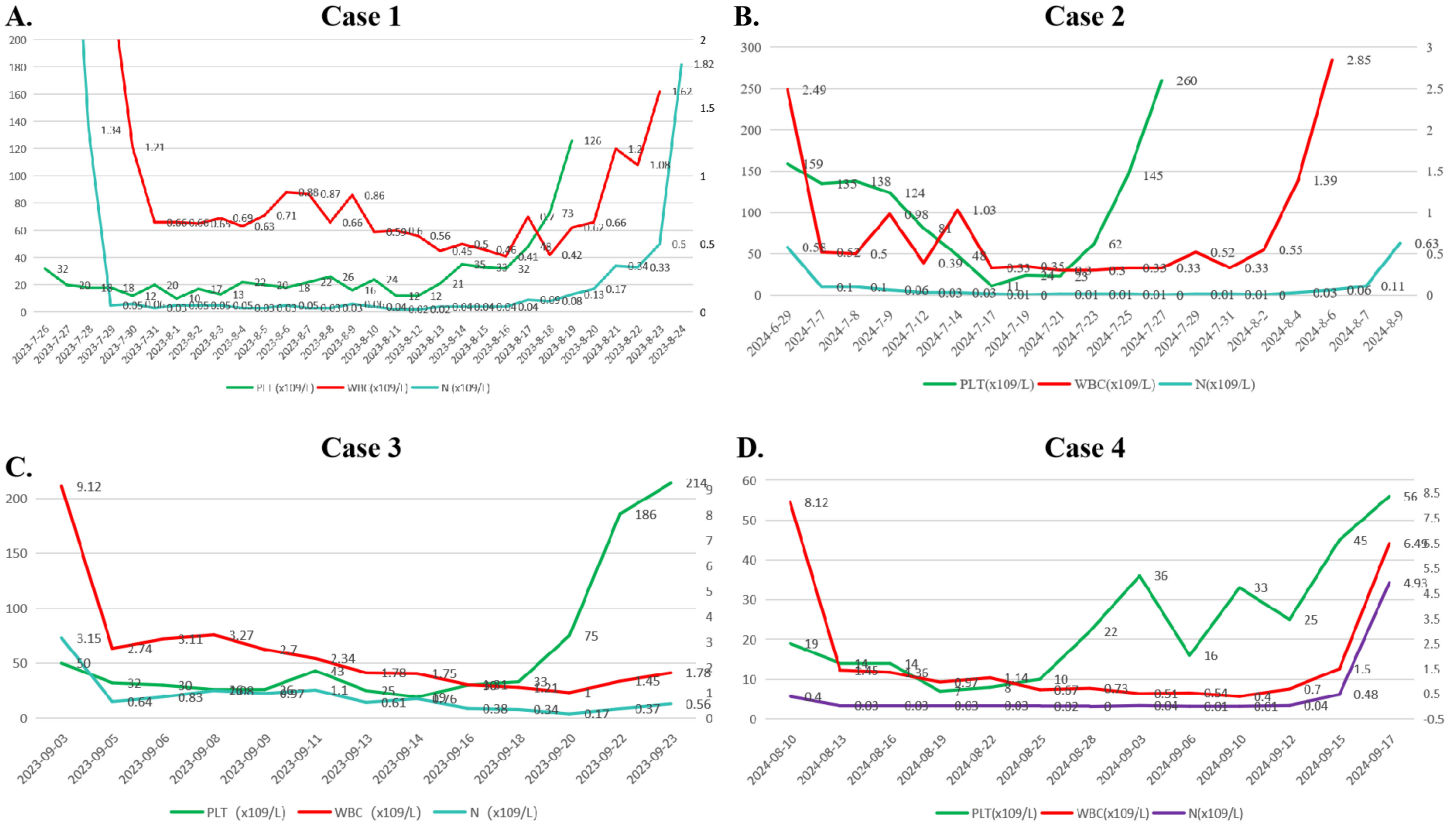
Changes in the hemograms of the 4 patients during induction therapy. **(A)** Case 1. **(B)** Caes 2. **(C)** Caes 3. **(D)** Caes 4. (Note: PLT, platelet; WBC, white blood cell; N#, neutrophil count).

A 43-year-old male was admitted to the hospital owing to fatigue on July 14, 2023. Analysis of the complete blood count revealed the following: white blood cell (WBC) count, 94.48×10^9^/L; hemoglobin (HGB) level, 67 g/L; platelet (PLT) count, 151 ×10^9^/L; and neutrophil count (N#), 0.95 × 10^9^/L. Analysis of the morphology of cells in a peripheral blood sample revealed that blast cells accounted for 85% of the total cells. Analysis of the morphology of the cells in a bone marrow (BM) sample revealed that proliferation was extremely active, with blasts accounting for 87.2% of the cells. BM immunophenotyping revealed that abnormal blasts accounted for 79.83% of the immune cells, which expressed HLA-DR, CD117, CD38, CD33, CD15, CD7, CD123, and cCD3 and weakly expressed CD3, CD13, CD56, CD2, CD64, cMPO, and CD11c. Karyotype analysis revealed a normal male chromosome pattern (46, XY)[20]. Next-generation sequencing (NGS) revealed mutations in CEBPA, NQO1, TNFAIP3, EZH2, KMT2D, and SETD2 and high expression of BCL-2, and no abnormal fusion gene was found. The patient was definitively diagnosed with T/myeloid MPAL. All diagnoses were made in accordance with the Chinese guidelines for the diagnosis and treatment of adult acute lymphoblastic leukemia (2021). The patient was infected with severe acute respiratory syndrome coronavirus 2 (SARS-CoV-2) and had a persistently high body temperature and severe lung infection, and his basic condition was poor. Since the patient was not eligible for receiving standard induction regimens, the VA regimen (ven 100 mg d1, 200 mg d2, 400 mg d3~28, azacitidine 100 mg, d1~7) was given to the patient on July 27, 2023, to induce remission. Analysis of the morphology of cells in a BM sample obtained after 15 days of treatment revealed 10.5% blast cells and measurable residual disease (MRD) detected by multiparameter flow cytometry (FCM), with 4.9% abnormal blasts (FCM analysis for all MRD assessments in this study was performed using the standardized 10-color panels with a sensitivity threshold of 0.01%). During treatment, the patient experienced complications such as a persistently high body temperature, severe lung infection, and pleural effusion (some images from the chest CT examination are shown in **Figure 1**). Morphological examination of a BM sample obtained on day 29 suggested CR. An analysis of MRD revealed no abnormal blast cells. Genetic testing revealed no mutations in the CEBPA gene. The first cycle of myelosuppression lasted 26 days. The changes in the hemogram are shown in **Figure 2**. The patient subsequently received 1 cycle of the VA regimen consolidation treatment again. After remission, the patient underwent lumbar puncture + sheath injection treatment and subsequently underwent matched sibling donor allo-HSCT. Unfortunately, the patient died of multiorgan failure due to capillary leak syndrome 2 days after transplantation.

## Case 2

A 51-year-old female was admitted to the hospital on May 24, 2024, for routine surveillance after a more than 2-year history of breast cancer treated with multiple cycles of chemotherapy (doxorubicin + cyclophosphamide). Analysis of the complete blood count revealed the following: WBC count, 1.57×10^9^/L; HGB level, 107 g/L; PLT count, 160×10^9^/L; and N#, 0.17×10^9^/L. Analysis of the morphology of cells in a peripheral blood sample revealed that blast cells accounted for 3% of the cells. BM aspiration was performed, and analysis of the samples revealed that 60% of the blood cells were blasts, 53% were positive for peroxidase (POX) staining, and 88% were positive for glycogen (PAS) staining. Leukemia immunophenotyping revealed that abnormal cells accounted for approximately 52.44% of the karyocytes, and the immunophenotype was mixed with predominant early myeloid differentiation and minor early B-lymphoid lineage involvement; the main proteins expressed were CD38, CD19, CD13, HLA-DR, CD34, CD117, and cMPO, and the cells partially expressed CD22 and cCD79a. Nucleated cell analysis revealed the following: 46,XX,i(7)(p10), t(11;17)(q23;q21)[4]/46,XX[7]. NGS revealed negative results, indicating that no fusion gene was present. The definitive diagnosis was B/myeloid MPAL. The patient had previously received multiple cycles of chemotherapy for breast cancer treatment and therefore refused to undergo intensive chemotherapy again. Therefore, on July 7, 2024, the VD regimen (venetoclax 100 mg d1, 200 mg d2, 400 mg d3~28, decitabine 25 mg, days 1~10) was used to induce remission. During treatment, except for transient neutropenia combined with fever, the patient did not complain of obvious discomfort. Examination of a BM sample obtained on day 22 revealed that proliferation was significantly active, with blast cells accounting for 0.6% of the cells. Flow cytometry analysis for MRD revealed early myeloid cells with abnormal expression of CD19, accounting for approximately 0.02% of the total cells. After one course of treatment, the efficacy evaluation revealed CR. The myelosuppression period of the first cycle lasted 21 days. The changes in the hemogram are shown in **Figure 2**. She subsequently received 1 cycle of the VD regimen consolidation treatment again. Allo-HSCT was recommended to the patient, but the patient and her family refused transplantation for financial reasons. The patient is currently receiving consolidation treatment.

## Case 3

A 40-year-old female was diagnosed with leukocytosis on August 11, 2023. At that time, her WBC count was 24.90×10^9^/L, her HGB level was 127 g/L, her PLT count was 211 × 10^9^/L, and her N# was 6.13×10^9^/L. Analysis of BM samples from other hospitals revealed that blast cells accounted for 53.5% of the total cells. Examination of a BM sample obtained after admission revealed that immature lymphoid cells accounted for 11.6% of the cells, and 43.3% of the cells were blasts and immature monocytic cells. BM immunophenotyping revealed that abnormal naive B lymphocytes accounted for 22.34% of cells (CD34, CD19, CD10, CD22, CD38, CD13, HLA-DR, CD33, cCD79a, and CD123), and abnormal myeloid blasts accounted for 11.6% of cells (CD34, CD117, HLA-DR, and MPO). NGS revealed no gene mutation; however, the fusion gene BCR::ABL1 (p190) was detected (BCR::ABL1/ABL1 (p190): 18.88%). Nucleated cell analysis revealed 46,XX, t(9;22)(q34;q11)[4]/46, idem, i(7)(q10)[6]. The patient was diagnosed with B/myeloid MPAL with BCR::ABL1. The patient was then given Ven and olverembatinib combined with azacitidine on September 4, 2023. The treatment regimens were as follows: Ven 100 mg d1, 200 mg d2, 400 mg d3~28, azacitidine 100 mg on d1~7, and olverembatinib 40 mg taken orally every other day. No blast cells were observed in the analysis of BM cell morphology at the 14-day follow-up. In the MRD analysis, abnormal blast cells accounted for 0.25%, and BCR::ABL1/ABL1 (p190) was found in 6.85% of the cells. Morphological analysis of a BM sample obtained on Day 28 revealed no blasts, MRD evaluation revealed no evidence of abnormal blasts, and BCR::ABL1/ABL1 (p190) was found in 3.39% of the cells. Morphological analysis of a BM sample obtained after the patient received one cycle of the original regimen revealed CR, MRD negativity, and BCR::ABL1/ABL1 (p190) negativity. The patient achieved complete molecular remission. The patient then underwent allo-HSCT, in which the patient’s sibling was the donor. Currently, more than 1 year after transplantation, the patient is still in MRD-negative CR.

## Case 4

An 80-year-old male was admitted to the hospital on August 6, 2024, owing to “pancytopenia for more than 2 months and fever for more than 2 hours”. A complete blood count was performed on May 28, 2024, because of dizziness, and the results were as follows: WBC count, 2.65×10^9^/L; N#, 0.91×10^9^/L; HGB level, 78 g/L; and PLT count, 36×10^9^/L. After symptomatic and supportive treatment, the patient’s dizziness was alleviated, and he was discharged from the hospital. The results from serial complete blood counts suggested pancytopenia. On August 6, 2024, the patient developed fever, with the highest body temperature being 39.5 °C; therefore, the patient was rehospitalized. The physical examination on admission revealed an anemic appearance, no petechiae or ecchymosis on the skin or mucous membranes, no palpable swelling of any of the superficial lymph nodes throughout the body, and no liver or spleen palpability under the costal margin. Complete blood counts revealed the following: WBC count, 1.99×10^9^/L; N#, 0.31×10^9^/L; HGB level, 37 g/L; and PLT count, 6×10^9^/L. Analysis of a peripheral blood smear revealed that blasts accounted for 45% of the cells observed. An examination of BM samples revealed active proliferation, with blast cells accounting for 78.4% of the cells observed. Flow cytometry immunophenotyping revealed that approximately 51.4% of the cells were myeloid blasts/immature cells. The immunophenotypes included CD117+, CD13+, CD33-, CD34+, HLDAR+, CD56-, CD19+ (minor subset), CD38+, CD36+ (partial), CD10-, CD3-, CD7-, CD5-, CD2-, CD14-, CD11b-, and CD15-, along with scattered weak positivity for cMPO. Additionally, approximately 22.6% of the cells were immature B-cell precursors, for which the immunophenotype was CD19+, CD22+, CD34+, HLDAR+, CD38+, CD13+, CD117-, or CD33-. These findings were in line with those of B/myeloid MPAL. An analysis of a BM biopsy sample revealed the hypercellularity of karyocytes with reduced granulocytes, erythroid lines, and megakaryocytic lineages, and immunohistochemistry of this sample revealed CD3- and scattered CD79a and MPO-, all of which are consistent with acute leukemia. Nucleated cell analysis revealed 92<4n>, XXYY[5]/46, and XY[12]. Leukemia fusion gene screening was negative, NGS revealed TET2(-), and the definitive diagnosis was B/myeloid MPAL. Owing to the advanced age of the patient, his family refused chemotherapy treatment; therefore, the patient began to receive induction therapy with Ven combined with an azacitidine regimen (Ven 100 mg d1-d21 and azacitidine 0.1 g d1-d5) on August 11, 2024. On August 27, BM examination revealed that the percentage of blast cells was 17.2%. MRD evaluation revealed that approximately 2.4% of the cells were myeloid blasts/immature cells, and approximately 0.3% of the cells were immature B-cell precursors. During the period of myelosuppression, the patient was given comprehensive treatment, such as anti-infective therapy and blood component transfusions. The myelosuppression period of the first cycle lasted 36 days. The changes in the hemogram are shown in **Figure 2**. An examination of a BM sample obtained on October 1, 2024, revealed 0.5% blasts, and normal hematopoiesis was restored, thereby meeting the criteria for CR. Follow-up analysis of a BM smear obtained on November 9, 2024, revealed active proliferation, with 0.8% blasts. MRD evaluation revealed no detectable immunophenotypically aberrant blast/immature cells. The patient is generally in good condition and has maintained continuous remission for more than 12 months, and the VA regimen is being administered regularly. The baseline clinical characteristics and post-treatment efficacy evaluations for the four patients are presented in **Table 1**.

**Table 1:** Clinical Baseline Workup and Efficacy Evaluation Following Treatment.

No.	Age (years)	Gender	WBC (×10^9^/L)	Bone marrow morphology	Flow cytometric immunophenotyping	Cytogenetics	Fusion genes	Gene mutations	Treatment regimen	Treatment response	allo-HSCT	OS (Months)
1	43	Male	94.48	Markedly hypercellular marrow with 87.2% blasts	Abnormal immature cells (79.83%) expressing HLA-DR, CD117, CD38, CD33, CD15, CD7, CD123, cCD3; weakly expressing CD3, CD13, CD56, CD2, CD64, cMPO, CD11c	46, XY[20]	Negative	CEBPA, NQO1, TNFAIP3, EZH2, KMT2D, SETD2	VA	CR with MRD(-)	Yes	4
2	51	Female	1.57	Hypercellular marrow with 60% blasts	Abnormal cells (52.44%) expressing early myeloid and early B-lymphoid immunophenotype: CD38, CD19, CD13, HLA-DR, CD34, CD117, cMPO+; partially expressing CD22, cCD79a	46,XX,i(7)(p10), t(11;17)(q23;q21)[4] /46,XX[7]	Negative	Negative	VD	CR	No	16
3	40	Female	24.9	Hypercellular marrow with 11.6% immature lymphocytes and 43.3% blasts/immature monocytes	Abnormal immature B-lymphocytes (22.34%): CD34, CD19, CD10, CD22, CD38, CD13, HLA-DR, CD33, cCD79a, CD123; Abnormal myeloid blasts (11.6%): CD34, CD117, HLA-DR, MPO	46, XX, t(9;22)(q34;q11)[4] /46, idem, i(7)(q10)[6]	BCR::ABL1 (p190)	Negative	Olverembatinib + VA	CR	Yes	25
4	80	Male	2.65	Hypercellular marrow with 78.4% blasts	Approximately 51.4% blasts/immature myeloid cells: CD117+, CD13+, CD33-, CD34+, HLA-DR+, CD56-, CD19+ (minor), CD38+, CD36+ (partial), CD10-, CD3-, CD7-, CD5-, CD2-, CD14-, CD11b-, CD15-, cMPO (scattered positivity); Approximately 22.6% blasts/immature B-cells: CD19+, CD22+, CD34+, HLA-DR+, CD38+, CD13+, CD117-, CD33-	92<4n>, XXYY[5]/46, XY[12]	Negative	TET2	VA	CR	No	12

WBC, White Blood Cell Count; CR, Complete Remission; MRD, Minimal Residual Disease; VA, Venetoclax + Azacitidine; VD, Venetoclax + Decitabine; allo-HSCT: Allogeneic Transplantation; OS, Overall Survival.

## Discussion

Members of the BCL-2 family of antiapoptotic proteins regulate the mitochondrial apoptosis pathway and are involved in the occurrence, development, metastasis and drug resistance of tumor cells (4). BCL-2 expression in AML cells is significantly higher than that in normal CD34+ hematopoietic stem cells, and this high expression mediates drug resistance in AML cells (5). As a potent oral Bcl-2 inhibitor, Ven has excellent antitumor effects on various leukemias (4, 6, 7). The combined use of Ven and HMA drugs has shown positive antileukemic activity in preclinical models and clinical trials (8). Currently, this combination therapy has been approved for the treatment of elderly or newly diagnosed AML patients with poor tolerance (8).

Additionally, Bcl-2 is highly expressed in immature T-ALL cells. *In vitro* studies have shown that Ven has a highly efficient antileukemic effect on T-ALL cell lines with high expression of Bcl-2. Further studies have shown that Ven has a highly efficient antileukemic effect on T-ALL cell lines with high expression of Bcl-2. There is synergy between Ven and cytarabine, doxorubicin, L-asparaginase, and dexamethasone (9). Therefore, the use of Ven may also be a new strategy for the treatment of T-ALL. The response rate of 12 patients with recurrent T-ALL who were treated with Ven combined with chemotherapy (including 3 patients treated with an HMA) reached 60% (10). Moreover, studies on B-ALL have shown that B-ALL with KMT2A rearrangement, hypodiploid B-ALL, or high expression of BCL-2 leads to the arrest of apoptosis in these B-ALL cells (11). An investigation of *in vitro* models revealed that Ven killed B-ALL cell lines and primary cells by inhibiting cell proliferation, inducing cell cycle arrest and inducing apoptosis, and the expression level of BCL-2 was closely related to the antileukemic effects of Ven on B-ALL cell lines (11). These findings suggest that the use of Ven may also be a new strategy for the treatment of B-ALL. In summary, Ven exhibited excellent antileukemic efficacy in both AML and ALL patients. These studies provide theoretical evidence for the use of Ven in the treatment of MPAL. In 2020, Wu et al. (12) reported the cases of two refractory T/myeloid MPAL patients who were given a VA regimen for induction therapy: 1 patient achieved CR, and 1 patient achieved no response (NR). In 2021, Wang et al. (13) reported the cases of two newly diagnosed B/myeloid MPAL patients. Both patients achieved CR after induction therapy with Ven combined with an HMA. For young AML patients at high risk according to the European Leukemia Network (ELN) recommendations, Xie et al. (14) administered Ven combined with decitabine, and the CR rate reached 93%.

Four newly diagnosed MPAL patients were included in this study. Patient 1 had T/myeloid MPAL and could not tolerate intensive chemotherapy owing to SARS-CoV-2 infection and lung infection after admission; therefore, the patient was given the VA regimen for 1 cycle and successfully achieved CR. Patient 2 had previously received multiple cycles of chemotherapy for breast cancer and refused to undergo another round of intensive chemotherapy for B/myeloid MPAL; therefore, the patient was given the VD regimen for 1 cycle and successfully achieved CR. Patient 3 had B/myeloid MPAL, was positive for BCR::ABL1, and was given the VA regimen. CR was successfully achieved with the combination of tyrosine kinase inhibitor (TKI) treatment for 1 cycle. Patient 4 was an 80-year-old man with B/myeloid MPAL who was unable to tolerate intense chemotherapy and was therefore given the VA regimen for 1 cycle. CR was successfully achieved. The myelosuppression period during induction therapy lasted 36 days. During the induction treatment for myelosuppression, Patients 1 and 4 developed concurrent infections (the infections in these patients were both controlled after active anti-infection and symptomatic treatments), whereas Patients 2 and 3 tolerated the treatment well and did not experience significant adverse effects.

In clinical practice, when managing patients with concomitant fungal infections, we preferentially opt for antifungal agents from the echinocandin or polyene classes to avoid drug-drug interactions. Should the use of a triazole antifungal be deemed necessary, it is our standard protocol to preemptively reduce the venetoclax dose, as recommended by prescribing guidelines, to mitigate the risk of overexposure. While therapeutic drug monitoring (TDM) for triazoles can be utilized to ensure their concentrations remain within the therapeutic range, we do not routinely monitor venetoclax plasma concentrations. This is supported by emerging evidence, including a population pharmacokinetic analysis by Brackman et al., which demonstrated no significant correlation between venetoclax exposure and efficacy or safety outcomes in treatment-naïve AML patients ineligible for intensive chemotherapy (15).

In the pre-Ven era, the main treatment regimens for MPAL were ALL-based regimens, AML-based regimens and “hybrid” regimens (ALL plus AML). One meta-analysis revealed that ALL-based induction therapy was superior to AML-based induction therapy, with a significantly higher complete hematological response (CHR) rate and a twofold reduction in the risk of death (16); however, there was no difference in 3-year overall survival (OS) between the two therapies (6.9% vs. 5.0%). This lack of long-term survival benefit despite higher initial response rates is likely attributable to the high relapse rate post-CR, which may be driven by the persistence of measurable residual disease (MRD) and resistant subclonal populations. On the other hand, the survival rate of the hybrid induction regimen is far lower than that of the other two regimens, possibly due to increased toxicity. The novelty of this study is that Ven in combination with HMAs, rather than traditional chemotherapy regimens, was used to treat 4 patients with newly diagnosed MPAL. The remission rate was high, and a good safety profile was observed. Notably, Patient 4, an 80-year-old male, has maintained continuous remission for over 12 months. After achieving CR, the duration of venetoclax administration in his consolidation therapy was strategically shortened to 10–14 days per cycle to balance efficacy and tolerability, demonstrating the feasibility of adapting this regimen for vulnerable elderly patients.

Our findings are consistent with and extend the growing body of evidence from case reports and series on the use of Ven-based regimens in MPAL. Similar to previous reports (3, 12, 13), our cohort—comprising both B/myeloid and T/myeloid subtypes—demonstrated high efficacy, with all four patients achieving CR after a single cycle of Ven+HMA. This aligns with the high CR rates observed in other small-scale studies. Regarding safety, the adverse events in our patients were primarily manageable myelosuppression and infections, a profile comparable to that reported in the literature for this combination and preferable to the significant toxicities often associated with intensive chemotherapy.

It is important to note the distinct clinical context of our study. Our patients were selected for Ven+HMA therapy due to specific contraindications to intensive chemotherapy, including advanced age, active SARS-CoV-2 infection, and significant comorbidities. This aligns with the real-world scenario where novel agents are often explored in vulnerable populations. While the 100% CR rate in this high-risk subgroup is highly encouraging, we acknowledge the limitations of our small sample size and the inherent selection bias. Therefore, the excellent efficacy and favorable safety profile observed here warrant further validation in larger, prospective cohorts that also include patients eligible for intensive therapies, to definitively establish the role of Ven+HMA in the frontline treatment landscape for MPAL.

In summary, our study demonstrated the effectiveness and feasibility of the use of Ven in combination with HMAs for the treatment of newly diagnosed MPAL patients, thereby providing a new treatment strategy for the treatment of MPAL.

## Data Availability

The original contributions presented in the study are included in the article/supplementary material. Further inquiries can be directed to the corresponding author.
